# *EDP2PDF*: a computer program for extracting a pair distribution function from an electron diffraction pattern for the structural analysis of materials

**DOI:** 10.1107/S1600576723004053

**Published:** 2023-05-31

**Authors:** Hongwei Liu, Keita Nomoto, Anna V. Ceguerra, Jamie J. Kruzic, Julie Cairney, Simon P. Ringer

**Affiliations:** a Australian Centre for Microscopy and Microanalysis, University of Sydney, Sydney, New South Wales 2006, Australia; b School of Aerospace, Mechanical and Mechatronic Engineering, University of Sydney, Sydney, New South Wales 2006, Australia; c School of Mechanical and Manufacturing Engineering, UNSW Sydney, Sydney, New South Wales 2052, Australia; SLAC National Accelerator Laboratory, Menlo Park, USA

**Keywords:** electron diffraction patterns, pair distribution functions, computer programs

## Abstract

Pair distribution function (PDF) analysis is a powerful technique to understand atomic scale structure in materials science. Unlike X-ray diffraction (XRD)-based PDF analysis, the PDF calculated from electron diffraction patterns (EDPs) using transmission electron microscopy can provide structural information from specific locations with high spatial resolution. The present work describes a new software tool for both periodic and amorphous structures that addresses several practical challenges in calculating the PDF from EDPs. The key features of this program include accurate background subtraction using a nonlinear iterative peak-clipping algorithm and automatic conversion of various types of diffraction intensity profiles into a PDF without requiring external software. The present study also evaluates the effect of background subtraction and the elliptical distortion of EDPs on PDF profiles. The *EDP2PDF* software is offered as a reliable tool to analyse the atomic structure of crystalline and non-crystalline materials.

## Abstract 2

A new program named *EDP2PDF* is developed for the conversion of an electron diffraction pattern into a pair distribution function for materials structure analysis.

## 1. Introduction

Diffraction techniques uncover the structures of solid-state crystalline materials via the classic Bragg diffraction equation (Bragg & Bragg, 1913 ▸). Bragg diffraction is traditionally applied to crystalline materials, but diffraction is physically valid for all materials, including gases, liquids, amorphous structures and quasicrystals. Structural features include the coordination number and short-range and medium-range ordering within a defined spherical radius in real space (Egami & Billinge, 2003 ▸). These features are related to the pair distribution function (PDF) that is extracted from experimental diffraction data using various radiation sources such as neutron, X-ray or electron beams.

The PDF can apply to many disciplines, including astronomy, physics, geography and materials science, as a structural analysis technique (Proffen *et al.*, 2003 ▸), including the study of metals and alloys, thin-film functional materials and carbon-based materials. An extensive review of the PDF method was given by Mitchell & Petersen (2012 ▸). The success of PDF extraction from an X-ray diffraction pattern is highly dependent on the type of material investigated. For example, embedded or dynamically created amorphous regions within thin films, powders in trace amounts or fine wires do not easily excite adequate X-ray diffraction intensity. Alternatively, Cockayne & McKenzie (1988 ▸) pointed out that the small wavelength of electrons and high beam intensity permit excellent resolution in real space. They developed a methodology for deriving PDF information from electron diffraction patterns (EDPs) of polycrystalline and amorphous thin films.

Several computer programs for PDF analysis have been published in the past decade. They were written to extract the total scattering structure factor and PDF from neutron powder diffraction profiles (Peterson *et al.*, 2000 ▸), X-ray diffraction patterns (Jeong *et al.*, 2001 ▸; Qiu *et al.*, 2004 ▸) and EDPs, such as *ProcessDiffraction* (Lábár, 2000 ▸), *SUePDF* (Tran *et al.*, 2017 ▸), *RDFTool* (Mitchell & Petersen, 2012 ▸), *eRDF Analyser* (Shanmugam *et al.*, 2017 ▸), *xPDF Suite* (Yang *et al.*, 2014 ▸) and *ePDF Tools* (Shi *et al.*, 2019 ▸; NanoMegas, Brussels, Belgium). The quantitative approach of electron PDFs is well summarized by Gorelik *et al.* (2019 ▸).

One common problem for the programs that use EDPs is background detection and subtraction from the extracted spectrum. For example, *SUePDF* (Tran *et al.*, 2017 ▸) asks users to choose two or three points from the baseline of the diffraction intensity profile to fit the background profile. However, there is a practical problem in choosing suitable key points from the baseline, which consequently suppresses the accuracy of background fitting. Furthermore, it requires the conversion of the EDP into an intensity profile using other programs. The MATLAB graphical user interface (GUI)-based program *eRDF Analyser* (Shanmugam *et al.*, 2017 ▸) is unable to provide a good solution to background subtraction for EDPs containing bright transmitted beams. *RDFTools* (Mitchell & Petersen, 2012 ▸) can create an intensity profile from an EDP, but it was developed as a script in the *Gatan Microanalysis Suite* (*GMS*), valid for Windows but not MacOS [although a software solution has been released that makes running *DigitalMicrograph* (32 bit) on Linux and MacOSX possible through the use of Wine (Hovden, 2012 ▸)]. A possible solution using current knowledge would be to develop an automatic strategy to extract the intensity profile and fully remove the background signal from the EDP. There are two strategies to correct the background: (i) in the original profile using a steep function, where Tran *et al.* (2017 ▸) introduced a function of 1/*x* ^2*n*^ type to model the background, or (ii) in the structure function or reduced structure function. All other programs follow the second strategy, even though non-structural information hidden in the structure function cannot be excluded if the original background is not subtracted from the original profile. Here, we follow the first strategy to detect background from the original profile but use a different background subtraction methodology, described in Section 4, and study how the background subtraction affects the structure function and final PDF profiles.

Another problem is the effect of the quality of the acquired image on the PDF profile. Modern EDPs are commonly recorded by a charge-coupled device (CCD) or complementary metal–oxide–semiconductor (CMOS) camera. The cameras must be calibrated to ensure no geometric distortion is observed in the acquired EDP image. However, geometric distortion is often present in EDP images due to instrument or alignment errors, as a result of non-orthogonality of the detector plane to the electron-beam axis of the transmission electron microscope, or possibly from astigmatism in the intermediate lens or in-column omega-filter lens if fitted. The effect of the image quality, *e.g.* ellipticity of diffraction patterns, on the PDF profiles has so far not been studied.

This article presents a computer program, *EDP2PDF*, to calculate an accurate PDF from electron diffraction rings. It addresses the above practical problems using crystalline gold nanoparticles and amorphous SiO_2_ glass. We demonstrate the data processing strategies used for both spectrum and image. We also discuss the effect of background subtraction and the ellipticity of the EDPs on the resulting PDF quality.

## 2. Program design

The *EDP2PDF* software deals with image and spectrum processing and includes several core modules that are important to output high-resolution PDFs. All the modules are based on crystallographic and mathematical algorithms found in many classic crystallography-related textbooks, and on centre- and line-detection algorithms as part of cognitive analysis techniques.

*EDP2PDF* enables users to alter multiple parameters for the conversion task, such as calibration of the raw EDP image, electron and X-ray wavelengths, sampling rate along the radius, and ranges of the scattering angle or vector. Other features include circle centre search, rectangle detection and estimation of the average atomic density of multiple-component materials.

*EDP2PDF* is of interest for studying a wide range of materials and has been used in recent studies of various bulk metallic glasses (Nomoto *et al.*, 2021 ▸, 2022 ▸; Best *et al.*, 2022 ▸).

## 3. Program requirements, functionality, availability and processing sequences

### 3.1. Requirements

*EDP2PDF* aims to provide a friendly GUI for PDF generation and can be run directly in Windows XP/7/10/11 operating systems (32 or 64 bit). Ten thousand lines of source code have been written using the object-oriented language Visual Basic 6.0 and compiled on the Microsoft platform using Microsoft Visual Studio 2008. The expected hardware configuration is as follows: more than 32 MB of RAM, 16 MB of HDD, and a Pentium M processor or higher. *EDP2PDF* has been successfully tested by independent users of Windows OS. However, we do not guarantee compatibility with the parallel Windows OS installed on a Boot Camp-enabled Macintosh computer.

### 3.2. Functions

The program has the following principal functions:

(i) It will convert an EDP into a PDF pattern using either automatic or manual mode.

(ii) It is valid for single-crystal or multi-crystalline electron diffraction.

(iii) Its conversion parameters can be adjusted as required.

(iv) It offers step-by-step conversion for non-complete EDPs.

(v) One-key conversion is available if scale bar information is correct.

(vi) The generated diffraction intensity profile and PDF pattern can be exported to ASCII plain text format.

(vii) Chemical information can be input manually, followed by saving in ASCII format, or can be imported from a saved ASCII file.

### 3.3. Availability

The software is free for academic purposes and available upon request from the correspondence author. A brief user manual is available as supporting information to this paper.

### 3.4. Procedure

The procedure covers a few major steps that are related to the experimental conditions, material chemical compositions and tunable extraction parameters. A routine procedure is described as follows:

(i) Input an EDP image in an acceptable format such as .jpg, .tif or .bmp.

(ii) Input the chemical compositions of the investigated materials for the calculation of average atomic structure factor and average atomic density.

(iii) Calibrate using either automatic searching or manual measuring.

(iv) Find the transmitted beam centre using either automatic searching or manual measuring.

(v) Set up conversion parameters for the radiation source wavelength, radial resolution and circular resolution, extracting the angle range.

(vi) Extract a diffraction intensity profile, with subsequent smoothing and SNIP background subtraction.

(vii) Normalize the diffraction intensity profile, and then generate the PDF and normalized PDF.

## 4. Modules and methods

### 4.1. Extraction of a diffraction intensity profile from an EDP

The program consists of image processing of an EDP in .jpg, .tif, .gif or .bmp format and the spectrum processing of a corresponding diffraction intensity profile extracted from the EDP. There are several basic aspects that affect the conversion quality. (i) An EDP could be obtained by a CCD/CMOS camera or scanned from a photograph. (ii) The raw data could be stored as greyscale or RGB colour. (iii) The intensity of the EDP could be inverted. (iv) The symmetry of the diffracted rings may not be perfect; the rings may be island-like or scattered rods, or the centre of the diffracted rings may be far away from the image centre. (v) The calibration of the EDP may be absent. These aspects commonly vary from one data set to another. Before the conversion, the image needs pre-processing so that it is suitable for data conversion.

All the methods for image processing (smoothing, sharpening, edge searching and binarization) and EDP processing (calibration, centre searching and conversion of diffraction intensity profile) are described in our previous paper on the related computer program *EDP2XRD* (Liu *et al.*, 2016 ▸). The following description focuses on spectrum processing via smoothing and background subtraction, the average atomic scattering factor, the structure function, and PDF normalization.

### 4.2. Spectrum smoothing and noise reduction

Spectrum processing of 1D data is required to smooth and denoise the extracted diffraction intensity profile to improve the outcomes of the background subtraction.

Most of the noise in electronic recording devices is correlated with random electron motion due to thermal agitation above absolute zero temperature (Boie & Cox, 1992 ▸). Smoothing removes the transformed signal components occurring at high values of the scattering vector *Q* in the transformed domain regardless of amplitude. Denoising removes small-amplitude components occurring in the transformed domain regardless of position. There are several methodologies for spectrum smoothing and denoising, such as the moving average algorithm, the least-squares or penalized least-squares method (Steinier *et al.*, 1972 ▸), Hankel matrix rank reduction (Fahmy & Hasan, 2004 ▸), wavelet transformation (Barclay *et al.*, 1997 ▸), and the Savitzky–Golay method (Savitzky & Golay, 1964 ▸). The Savitzky–Golay method is chosen in this work because it is preferred for a spectrum with a medium or high signal-to-noise ratio (SNR). It performs a least-squares fit of a small set of consecutive data points to a polynomial and takes the calculated central point of the fitted polynomial curve as the new smoothed data point.

**Figure 1:**
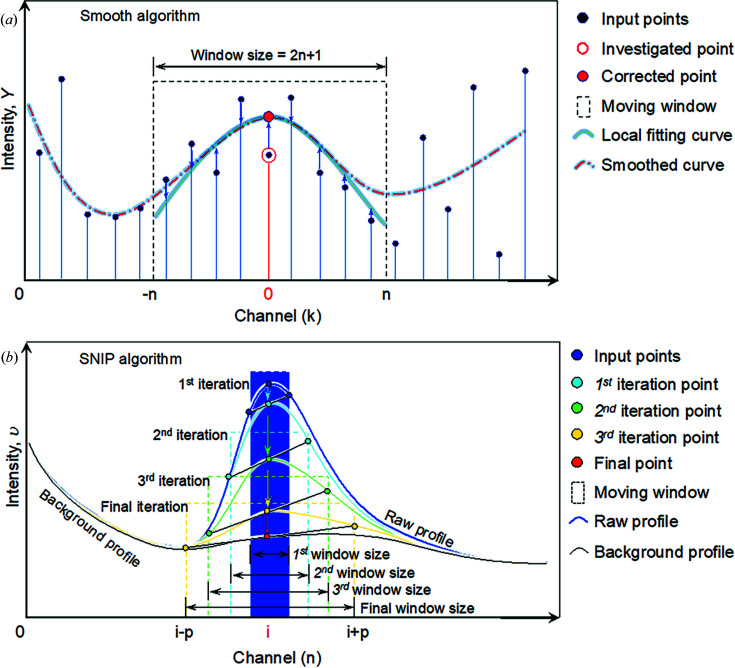
Spectrum processing algorithms for (*a*) smoothing by the Savitzky–Golay method and (*b*) background removal via the SNIP method.

Following this method, a group of integers (*A*_−*n*_, *A*_−(*n*−1)_,…, *A*_*n*−1_, *A_n_*) with a window size 2*n* − 1 were used as the weighting coefficients for the smoothing operation, as shown in Fig. 1 ▸(*a*). The use of these weighting coefficients, known as convolution integers, is the equivalent of fitting the data to a polynomial. The smoothed data point (*Y_k_*)_s_ from the Savitzky–Golay algorithm is given by

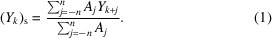

Here, *n* defines the moving window size (2*n* + 1), *A_j_* is the *j*th convolution integer of *A*(*n*), *Y_k+j_* is the *j*th value of the moving window and (*Y_k_*)_s_ is the final average value for the window with its centre located at *Y_k_*. The convolution integers depend on the filter width and the polynomial degree. For a diffraction intensity profile, the filter width can be 7 (*n* = 3) and the corresponding convolution integers *A*(3) are (−2, 3, 6, 7, 6, 3, −2).

### 4.3. Spectrum background subtraction

Contributions to the EDP data background include the direct-beam tail, energy-loss continuum and incoherent multiple scattering (Mitchell & Petersen, 2012 ▸). The radially symmetric component is much easier to correct in the integrated 1D profile, while all non-radial symmetric features in the background should be corrected prior to the integration.

The background information contains an irregular baseline profile, which is not easy to simulate, fit and remove accurately. Some published software such as *SUePDF* adopts a manual way of fitting the baseline based on the position of a few key points along it. The *ePDF Suite* currently uses a similar background subtraction to *PDFGetX3* (Juhás *et al.*, 2013 ▸). The present authors use a modification of Tran’s strategy, as mentioned in the *Introduction* [Sec sec1].

Both instrumental and computational methods have been proposed to detect and remove the background of a spectrum. The energy-loss effect can be suppressed by applying energy filtering, although this will induce a certain distortion such as filter lens aberration, which may not be easily detected, identified or corrected. Computational methods including first and second derivatives (O’Grady *et al.*, 2001 ▸), frequency-domain filtering (Mosier-Boss *et al.*, 1995 ▸), wavelet transformation (Zhang *et al.*, 2010 ▸), optimization (Beier & Berger, 2009 ▸), or polynomial fitting (Brennan *et al.*, 1997 ▸) are utilized in many application fields. Our program makes use of the sensitive nonlinear iterative peak-clipping (SNIP) algorithm (Morháč *et al.*, 1997 ▸) to remove background because it can achieve a fast calculation speed and less distortion, and possibly eliminates the background.

To enhance small peaks in a diffraction intensity profile, it is appropriate to apply the LSR operator (twice log operators plus square-root operator) before employing the SNIP algorithm:

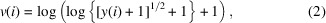

where *i* is the spectrum signal channel and *y*(*i*) is the count in channel *i*.

From *v*(*i*) we calculate, step by step, the vectors *v*_1_(*i*), *v*_2_(*i*) up to *v_m_* (*i*), where *m* is the iterator and a given free parameter and defines the number of steps of the SNIP algorithm [see Fig. 1 ▸(*b*) for an example of four-iteration processing]. For an X-ray diffraction pattern or diffraction intensity profile, the free parameter *m* can be half of the measured major peak channel width (or full width at half-maximum). The new value of channel *i* in the *p*th iteration step [ 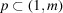 ] is

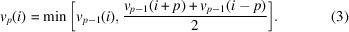

The inverse LSR operator is applied to obtain the resulting baseline spectrum *b*(*i*) after the vector *v_m_* (*i*) is calculated.

We point out that the C language code for SNIP on page 3 in the article by Morháč *et al.* (1997 ▸) contains some typographical errors. Readers will not obtain a rational result if this code is implemented verbatim. In particular, the two lines with the same sentence for (i = p; i < N − p; i++){ should be modified to for (i = p + m; i < N − p; i++){ in two locations. The correction vector *b*(*i*) is the detected baseline spectrum, which is then subtracted from the original vector *y*(*i*) to produce net peaks with no background. Here *b*(*i*) is the background count at the spectrum signal channel *i*.

### 4.4. PDF generation

The extracted diffraction intensity profile is identical to total scattering containing both Bragg peaks and diffuse scattering intensities over a wide range of scattering vector magnitude *Q*. *Q* has the form

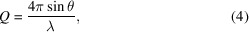

and the reader is referred to Appendix *A* [App appa] for a full description of the scattering vector magnitude.

The atomic scattering factor of X-rays is different from that of electrons. The relationship between them can be obtained by the Mott–Bethe formula (Kirkland, 2010 ▸),

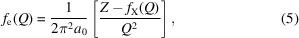

where *Q* is the scattering vector magnitude defined in equation (4) and cannot be zero, *Z* is the atomic number, *a*_0_ is the Bohr radius (0.5292 Å), and *f*_X_(*Q*) and *f*_e_(*Q*) are the atomic scattering factors for X-rays and electrons, respectively. The normalized scattering curves are fitted to a nine-parameter equation developed by Cromer & Mann (1967 ▸). Knowing the nine coefficients, *a_i_*, *b_i_* and *c*, and the wavelength λ, we can calculate the scattering factor for each atom at any given scattering angle,

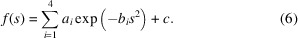




Note that a different scattering vector magnitude *s* defined by

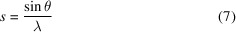

is used only for the atomic scattering calculation in equation (6). The relationship between *Q* and *s* is 

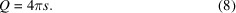




In reciprocal space, the observed sharp Bragg peaks are assigned to long-range ordering in the investigated crystal structure. In contrast, strong diffracted peaks are not visible in amorphous materials, which usually generate weak scattering signals. What is interesting is that the structure related to short-range order, between disorder and long-range order, leads to diffuse diffraction, although this is isotropic in all azimuthal directions. The difference between the ideal scattering and the raw experimental data can be used to calculate the total scattering structure function *S*(*Q*) defined by

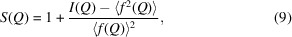

where *I*(*Q*) is the experimental diffraction intensity without background signal, and 〈*f* ^2^(*Q*)〉 and 〈*f*(*Q*)〉^2^ are the square mean and mean square, respectively, of the atomic scattering factors defined by

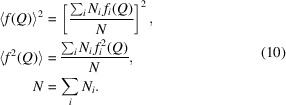

Here, *N* is the number of elements in the investigated material and *N_i_* is the atomic percentage of the *i*th element. *S*(*Q*) oscillates about 1 at high frequency or high *Q* because there is at least one atom that could be found at a distance corresponding to a high *Q* value from the investigated atom.

The reduced structure function (Petkov *et al.*, 2005 ▸) has the form

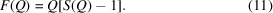

Reducing the structure function means that *F*(*Q*) oscillates around 0 at high *Q* values.

The PDF is deduced by applying a Fourier transformation to the reduced structure factor *F*(*Q*) (Bednarcik *et al.*, 2011 ▸; Farrow & Billinge, 2009 ▸),

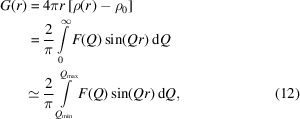

where ρ(*r*) is the local atomic number density, ρ_0_ the average number density of the investigated sample (see Appendix *B* [App appb] for details) and *r* the radial distance.

The convolution in equation (11) induces some artefact peaks at low and high values of *r* when it terminates on both sides of the scattering vector *Q*_min_ and *Q*_max_. This is equivalent to low- and high-frequency noise. Low-frequency noise is often removed by a linear fitting of the *G*(*r*) profile in the range (0, *r*_min_).

### 4.5. Normalized pair distribution function

The PDF may contain signals which are not physically correct. To obtain a PDF that has a probability density of finding a pair position physically and mathematically, the PDF is normalized according to the average atomic number density. If the resulting average atomic number density is not correct, the normalized PDF will cross the zero point of the profile’s *y* axis. Appendix *C* [App appc] contains more details about the extraction of the average coordination number.

The normalized PDF *g*(*r*) represents the probability density, or in other words, the probability of finding a pair position at a distance *r* in spherical space. The normalized PDF is derived from the untreated PDF as follows (Kodama *et al.*, 2006 ▸; Gilbert, 2008 ▸; Tran *et al.*, 2016 ▸):

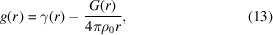

where γ(*r*) is equal to the nanoparticle form factor [γ(*r*) = 1 for a bulk sample] and ρ_0_ is the average atomic number density. The normalized PDF (i) must be positive [*i.e.* *g*(*r*_min_) > 0] when *r* ≥ *r*_min_, (ii) should be zero when *r* < *r*_min_, and (iii) must be unity [*i.e.* *g*(*r*_max_) = 1] when *r* ≥ *r*_max_.

### 4.6. Experiment and materials

Two typical crystalline and amorphous specimens were used for the EDP experiments and PDF analyses. A commercial calibration sample, namely a gold shadowed latex standard on a 200 mesh copper grid (S016), purchased from ProSciTech Company, was chosen as the crystalline material. This specimen has a moderate to heavy gold coating, applied using shadowing techniques, on latex microspheres. These latex microspheres have a diameter of ∼0.20 µm. The microspheres are all supported on a carbon-coated Formvar film on 3.05 mm 200 mesh copper grids. The supporting Formvar is approximately 10 nm thick with 5–10 nm of carbon film. The gold nanoparticles are spherical in shape with a mean diameter of around 5 to 20 nm.

High-quality fused quartz (Apricus Company) was used for amorphous sample testing. Fused quartz is the amorphous form of quartz, chemically known as SiO_2_. It is a quartz with extremely low concentrations of impurities, made by purifying and melting natural crystalline quartz, usually natural quartz sand. A transmission electron microscopy (TEM) sample was prepared using standard Tripod polishing to obtain an observation area with a thickness less than 100 nm.

The electron diffractograms were recorded at room temperature using a double aberration-corrected transmission electron microscope (Themis Z, Thermo Fisher Scientific) operated at 300 kV acceleration voltage with a Ceta 16 M CMOS camera. A selected-area aperture size of 40 µm and a camera distance of 285 mm were used for selected-area diffraction. The diffraction pattern images were recorded simultaneously in both .dm3 and .tif formats. The PDF calculation is based on EDPs with *Q*_max_ = 20 Å^−1^ and *Q*_min_ = 1.0 Å^−1^.

## 5. Main functions and features

This section describes the software implementation of the theory, using the gold nanoparticle sample as an example.

### 5.1. EDP conversion into diffraction intensity profile

**Figure 2:**
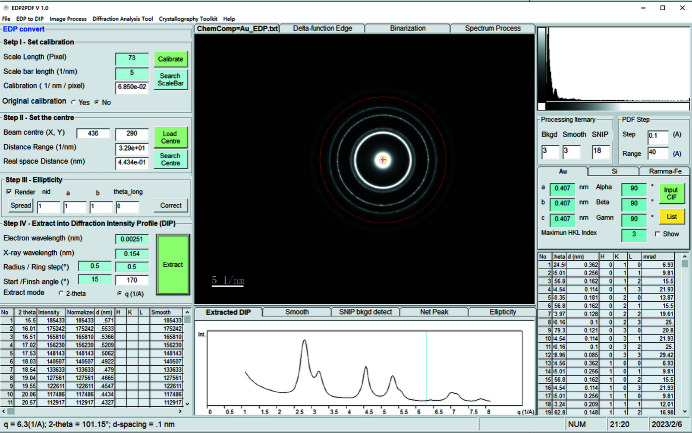
A screenshot of the software *EDP2PDF* working on converting an EDP into a diffraction intensity profile of an Au nanoparticle sample.

This part of the function is the same as the previously developed software *EDP2XRD* (Liu *et al.*, 2016 ▸), which allows users to input a 2D image file of an EDP collected from the target material and extract it into a diffraction intensity profile as a 1D spectrum. The resulting spectrum is then easily processed for further analysis. Fig. 2 ▸ is a screenshot of *EDP2PDF*. A small electron wavelength (0.085 nm) was chosen to extract a diffraction intensity profile in order to cover *Q* values as high as possible. The default image format for processing is 8 bit greyscale .tif. Other formats, including .jpeg, .bmp and .gif, are also available for direct input. Although the default image format is 8 bit, a 16 bit image also works. However, other formats including .dm4, .dm3, .emi/.ser and .mrc are not currently supported. We are keen to include them in an upgraded version in the near future.

### 5.2. Spectrum smoothing and background subtraction

**Figure 3:**
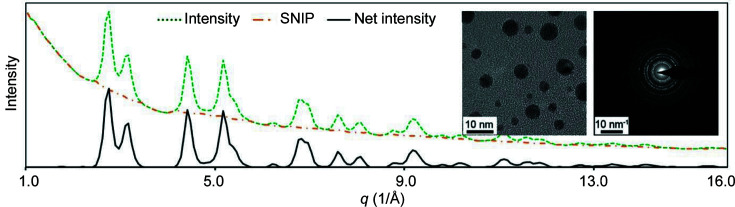
Processing of the extracted diffraction profile on gold nanoparticles (inset, left). The experimental spectrum (green dashed curve) was derived from the EDP (inset, right). It was then smoothed and its background (orange dashed–dotted curve) was detected by the SNIP algorithm. The final background subtraction gives the net peaks (black solid curve).

Fig. 3 ▸ shows the extracted diffraction intensity profile (green dashed curve) using the gold nanoparticles (inset, left) and the EDP (inset, right). The spectrum is then smoothed to remove sharp noise by applying an average moving window algorithm (Savitzky & Golay, 1964 ▸). On the smoothed spectrum base, the SNIP algorithm is applied to detect the background (orange dashed–dotted curve) and obtain the net peaks (the black solid curve), as shown in Fig. 3 ▸.

### 5.3. Chemical composition editor and number density estimation

**Figure 4:**
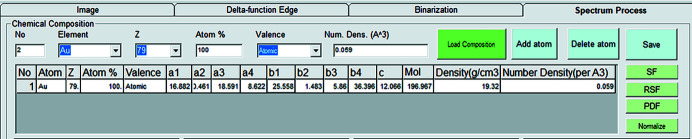
The chemical composition input interface to show all the elements, the corresponding atomic number, atomic percentage and nine-parameter fitting data. For convenience of estimation of the average atomic number density, the molar mass and mass density are provided. The atomic number density for each of the elements is estimated using the molar mass and mass density, and the average atomic number density is output.

The sample’s chemical composition is required to generate a proper PDF from the net peaks. The program provides a simple user-friendly interface to assist data input. The interface is shown in Fig. 4 ▸.

Users must input the atom type, atomic percentage and valence states. The atomic number for each element will be generated automatically. To aid with the calculation of the atomic scattering factor using equation (6), a chemistry input tool includes a database containing the nine fitting parameters for 92 elements, including their various valence states.

For a given material with *N* elements, each has an atomic number *Z*(*r*) (*r* = 1 to *N*), a molar mass *M*(*r*), a density ρ(*r*) and an atomic percentage *C*(*r*). The average atomic number density ρ is calculated using

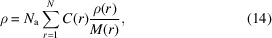

where *N*_a_ is Avogadro’s number (6.022 × 10^23^).

The calculation of the average atomic number density for certain chemistries is automatic if the elements present, their valences and their atomic percentages are provided. The nine-parameter fitting and average atomic number density estimation are then displayed automatically by clicking the ‘Save’ button in the user interface.

As to determination of the number density from PDFs calculated by EDPs, it is not usually easy because of the lack of structural information such as number density, the multiple scattering present in the data and the *Q*_max_ truncation effect at small *r*.

### 5.4. PDF conversion with editable parameters

**Figure 5:**
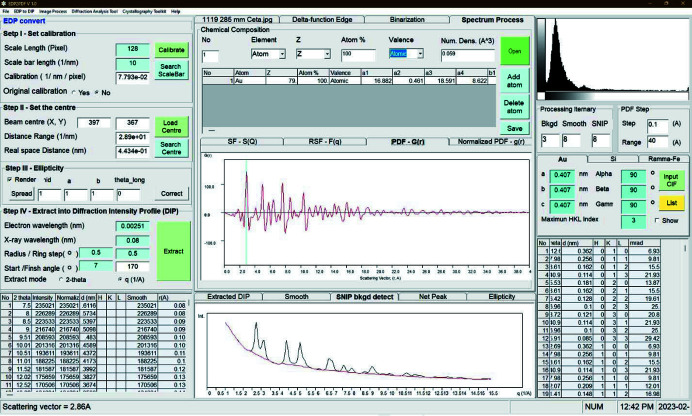
A screenshot of the software *EDP2PDF* working on extracting PDF and normalized PDF profiles from the calculated diffraction intensity profile of the nanocrystalline Au sample.

Fig. 5 ▸ shows the user interface to extract the PDF and normalized PDF profiles. The PDF of the Au nanoparticle sample was calculated from the EDP shown in Fig. 2 ▸ via the steps in Section 4.4. The final normalized PDF, obtained using the normalization method described in Appendix *C* [App appc] and the average atomic number density estimated by utilizing equation (14), is shown as a red curve in the ‘Normalized PDF – *g*(*r*)’ tab in Fig. 5 ▸. Data with *Q* values of less than 1 Å have been omitted.

**Figure 6:**
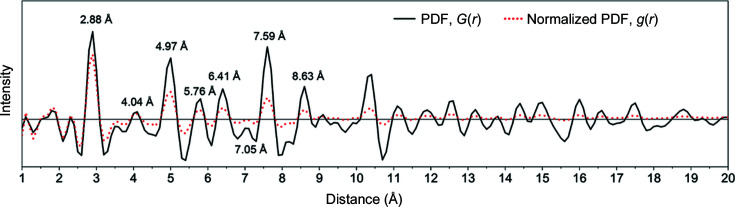
The PDF and the normalized PDF of the Au nanoparticle sample. Note that the peaks are identical to the Au crystal interatomic distances.

Fig. 6 ▸ presents physically meaningful peaks observed from the PDF and the normalized PDF profiles. The profiles peak at 2.88, 4.04, 4.97, 5.76, 6.41, 7.05, 7.59 and 8.63 Å in short-range order, as labelled in Fig. 6 ▸. These peaks are in good agreement with a PDF measured on a similar sample of gold nanoparticles with an average size of 3 nm suspended in water whose PDF was converted from synchrotron X-ray diffraction data using Monte Carlo simulation (Petkov *et al.*, 2005 ▸). Since the Au particles were prepared on a carbon-coated TEM grid, the signals at ∼1.4 and 2.3 Å could be associated with the aromatic first and second C—C inter­atomic distances, respectively (Majid, 1983 ▸), and that at 3.6 Å with the distance between the graphene sheets in carbon. The signal at ∼1.8 Å is probably artificial, although it is close to the Au—C interatomic distance (Benitez *et al.*, 2009 ▸), as a significant concentration of Au—C bonds should not be seen in the PDF.

It may be noted that the PDF profiles in Figs. 5 ▸ and 6 ▸ are not smooth. It seems this is due to insufficient data points. The quality of the generated PDF curves is correlated with the angular resolution along the radial and tangential directions of an EDP during extraction into an electron diffraction intensity profile.

## 6. Application to crystalline and amorphous materials

### 6.1. Au nanocrystals

**Figure 7:**
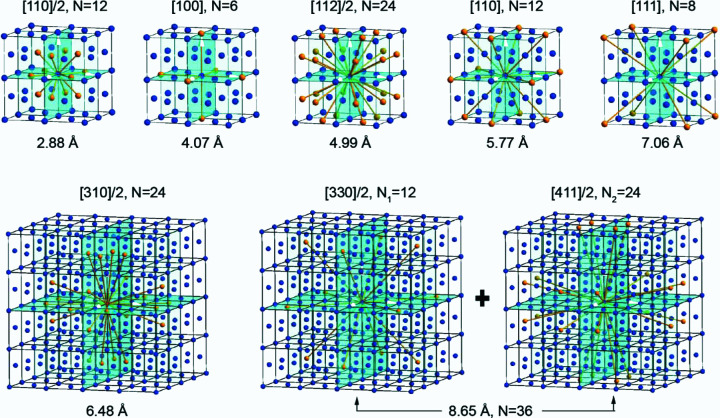
Three-dimensional atomic configurations for the different coordination numbers with atomic distance, covering the first seven peaks in the normalized PDF profile shown in Fig. 4 ▸. Note that for the coordinate peak at 8.65 Å the coordinate number 36 is the sum of two different interatomic distances, [330]/2 and [411]/2.

The PDF profile of a nanocrystalline Au sample in Fig. 6 ▸ indicates the peaks in the profile occupying a certain inter­atomic distance. By investigating the face-centred cubic structure of Au and measuring the distance between two atoms in the Au cell which has a known lattice parameter *a* = 4.078 Å, the peaks corresponding to the interatomic distances are assigned as shown in Table 1 ▸. The three-dimensional atom configuration is shown in Fig. 7 ▸. The blue spheres in each of the panels in Fig. 7 ▸ are Au atoms. These coloured Au atoms share the same interatomic distance, which is identical to the interatomic distance measured using the PDF profile, indicating the accuracy of the *EDP2PDF* software. The validation of this approach using a nanocrystalline Au sample indicates that it is suited to application to other crystalline and amorphous samples.

### 6.2. Amorphous silica

**Figure 8:**
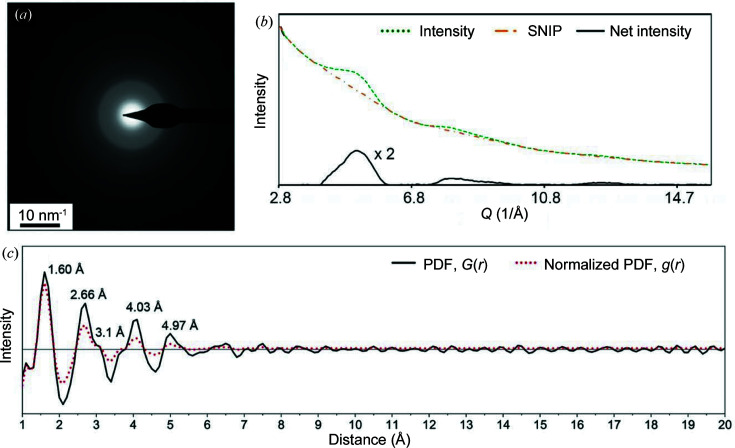
(*a*) The electron diffraction pattern, (*b*) the extracted diffraction intensity profile and its noise-filtered pattern, and (*c*) the corresponding PDF and the normalized PDF of the glassy silica sample. Note that the peaks in the PDF are identical to the Si—O, O—O and second Si—O bonding distances with increasing length order in direct space.

Glassy or amorphous silica (α-SiO_2_) is a typical amorphous solid with isotropic properties suited to many industrial and scientific applications. The building block of glassy silica is a tetrahedral SiO_4_ unit where one silicon atom occupies the centre of the tetrahedron and four oxygen atoms comprise the corners. The conventional sharp Bragg diffraction reflections for ordered silica disappear for the amorphous state with only a broad diffuse diffraction disc remaining. We used high-purity fused quartz in the form of a tube with very small amounts of impurities (alkalis, Fe_2_O_3_, TiO_2_, MgO and ZrO_2_) (Platias *et al.*, 2013 ▸) as an amorphous structure test case. Fig. 8 ▸(*a*) is a typical EDP for the glassy silica sample, consisting of a diffraction ring with a very broad width in reciprocal space. Fig. 8 ▸(*b*) shows the corresponding diffraction intensity profile extracted from the EDP in Fig. 8 ▸(*a*). The original scattering curve (dashed green curve) shows very weak and broad peaks while the background noise level is relatively high. The net peak profile (solid black curve) was produced using a standard SNIP approach to remove the background intensity (dashed–dotted orange curve). The final diffraction intensity profile shows only two broad peaks at about 25 and 84°, corresponding to *Q* values of 1.76 and 5.46 Å^−1^, respectively.

This diffraction intensity profile gives a very clear PDF curve [Fig. 8 ▸(*c*)]. The first five peaks at 1.60, 2.66, 3.1, 4.03 and 4.97 Å correspond to the interatomic distances of atomic pairs of Si—O^I^, O—O^I^, Si—Si, Si—O^II^ and O—O^II^, respectively. These are close to the reported short-range order peaks of a random network model for silica (Dove *et al.*, 1997 ▸; Van Ginhoven *et al.*, 2005 ▸; Vollmayr *et al.*, 1996 ▸), giving reported values of 1.61, 2.63 and 3.9 Å, or for vitreous silica (Mozzi & Warren, 1969 ▸), giving 1.62, 2.65, 4.15 and 4.95 Å. A minor peak for the Si—Si distance at 3.1 Å (Biswas *et al.*, 2018 ▸) is also observed in the PDF curve, which may be assigned to an artificial peak due to *Q*_max_ clipping. However, when *Q*_max_ increases to 40 Å, the peak at 3.1 Å disappears. The peak at 3.6 Å is due to the carbon supporting film.

## 7. Discussion

### 7.1. The effect of background on the PDF

When recording an EDP, it is common to collect background information such as the count of inelastic and incoherent scattering electrons, the CCD or CMOS camera gain pattern, and dark current. This background noise can be suppressed by various methods, including energy filtering to remove inelastically scattered electrons, and removing gain reference and dark reference by the camera. However, despite the best efforts at background minimization, it is still necessary to remove background signals from the extracted diffraction intensity profile. The SNIP methods described in Section 4.3 provide an effective means of background subtraction so that we can evaluate how the background signal on the extracted diffraction profile affects the final PDF profile.

**Figure 9:**
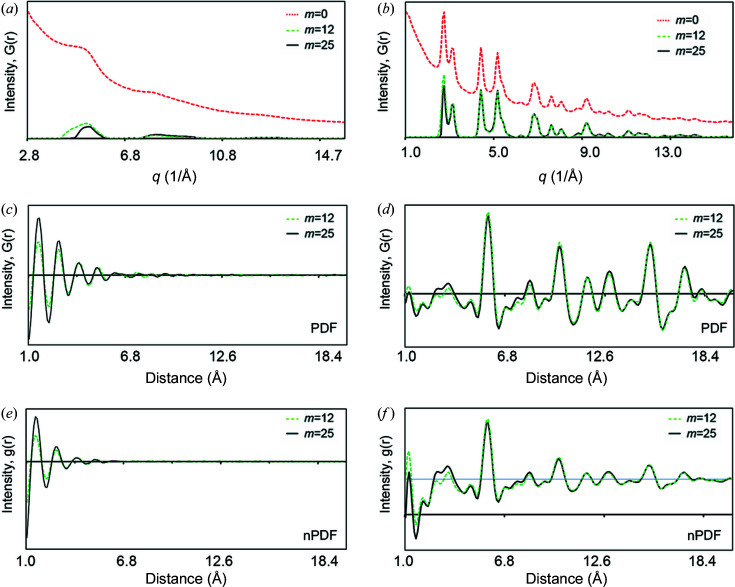
The effect of background on the quality of the PDF with various SNIP background subtraction iterations (*m* = 0, 12 and 25). Diffraction intensity profiles are plotted for (*a*) amorphous silica and (*b*) Au, followed by extraction into PDFs of (*c*) the amorphous silica and (*d*) the Au sample. The normalized PDFs are shown for (*e*) amorphous silica and (*f*) the Au sample.

Fig. 9 ▸ compares the effect of background information on the resulting PDF for Au nanoparticles and amorphous silica. We tested three SNIP background subtraction levels (the iteration process *m* values are 0, 12 and 25, as described in Section 4.3). The PDFs were converted with a full background (*m* = 0), without background subtracted using *m* = 25, and with a moderate background subtracted using *m* = 12. For the amorphous silica sample [Figs. 9 ▸(*a*) and 9 ▸(*b*)] there are significant differences in the peak positions, indicating that the high residual background of the converted diffraction intensity profile with *m* = 0 generates pseudo-scattering peaks in the PDF. The PDF peak positions with *m* = 12 are similar to those with *m* = 25, but the peak positions deviate from their theoretical values (*e.g.* the Si—O^I^ peak at 1.43 Å), indicating that the background subtraction is insufficient. For the Au nanoparticles [Figs. 9 ▸(*c*) and 9 ▸(*d*)], the PDF peaks using *m* = 0 show a higher noise level than those using *m* = 12 and 25. The other peaks also differ slightly from each other and some pseudo-scattering peaks are generated. The PDF profiles with *m* = 12 and 25 show that most of the major scattering peaks are quite similar, indicating that the background subtraction is sufficient. These results lead us to believe that our SNIP method works effectively to remove undesired background noise and improve the PDF accuracy.

### 7.2. Evaluation of the SNIP method for different thicknesses of amorphous silica

**Figure 10:**
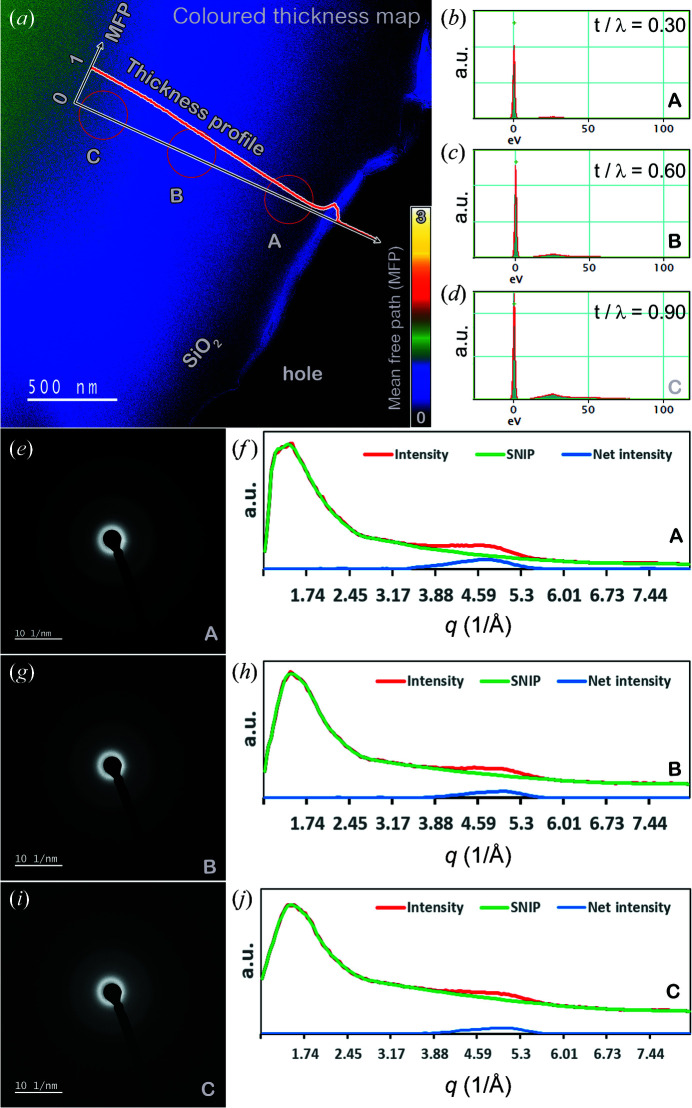
The effect of thickness on the SNIP method for background subtraction. (*a*) A coloured thickness map of the amorphous silica thin film. (*b*)–(*d*) ZLPs for the different thickness areas A, B and C, annotated with the ratios of MFPs. (*e*), (*g*) and (*i*) EDPs of the areas A, B and C, respectively. (*f*), (*h*) and (*j*) SNIP processing on the extracted diffraction intensity profiles for each area.

The SNIP method for background detection from an extracted diffraction intensity profile has been evaluated using different samples. To examine the robustness of this method further, sample thickness is considered as a practical issue affecting the data. We collected EDPs from amorphous silica at three positions with increasing thickness and a gap of 20 nm. The thickness is estimated using the electron energy-loss spectrometry technique based on the mean free path of amorphous silica. The analysis is shown in Fig. 10 ▸.

Fig. 10 ▸(*a*) shows a thickness map of the amorphous silica thin film rendered with pseudo-temperature colour. The plasmon spectrum is routinely used to measure the thickness of a TEM specimen because more plasmons are excited as the electrons go through a thicker specimen. If diffraction effects are negligible, the thickness of a TEM sample can be evaluated as a multiple of the inelastic mean free path (MFP) using the log-ratio method. The thinner the sample, the smaller the log-ratio value. The thickness profile in terms of the ratio to MFP is plotted in Fig. 10 ▸(*a*) and the three selected areas of different thicknesses are marked A, B and C. The zero-loss peaks (ZLPs) for each area are plotted in Figs. 10 ▸(*b*)–10 ▸(*d*), annotated with the measured log-ratio value. The selected-area diffraction patterns for each area are shown in Figs. 10 ▸(*e*), 10 ▸(*g*) and 10 ▸(*i*). The results of extraction into diffraction intensity profiles followed by SNIP background detection are seen in Figs. 10 ▸(*f*), 10 ▸(*h*) and 10 ▸(*j*). This shows that the SNIP method can detect background information from all three diffraction intensity profiles and give the same net diffraction peak. This is a good example showing the robustness of the SNIP method in terms of sample thickness variation.

### 7.3. Ellipticity of EDPs

**Figure 11:**
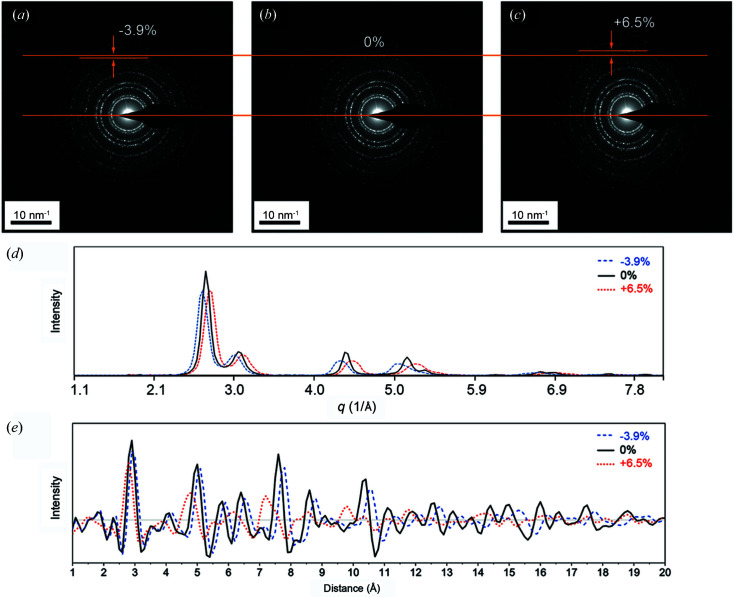
The effect of the ellipticity of an EDP of Au nanoparticles on the resulting diffraction intensity profile and PDF patterns. (*a*)–(*c*) EDPs with ellipticities of −3.9, 0 and 6.5%, respectively. (*d*) The extracted diffraction intensity profile, showing that the diffracted peaks shift to the left for a negative elliptical distortion. (*e*) The resulting PDF patterns and the peak shifts, which are dependent on the ellipticity.

This section describes the effect of an EDP’s ellipticity on the extracted diffraction intensity profile, and consequently on the derived PDF, using the example EDP of Au nanoparticles. Lábár (2000 ▸) published a pioneering program named *ProcessDiffraction* with the capability of ellipticity correction. Mitchell & Van den Berg (2016 ▸) developed a *DigitalMicrograph* script package called *Ellipse Fitting Analysis* (*EFAnalysis*, Version 1.0) to detect and correct the ellipticity of EDPs. Figs. 11 ▸(*a*), 11 ▸(*b*) and 11 ▸(*c*) show EDP patterns induced with artificial degrees of ellipticity of −3.9, 0 and 6.5%, respectively.

Ellipticity causes broadening and splitting of the peaks in the integrated profile. The peak broadening is asymmetric, which can make it appear as if the profile is expanding or contracting. The deviation from a perfect circle is highlighted in each of the EDP images. After converting to a diffraction intensity profile in Fig. 11 ▸(*d*), the peaks shift asymmetrically to a lower angle for −3.9% or a higher angle for 6.5% to an extent that is roughly proportional to the degree of ellipticity. Accordingly, the EDP gives a PDF with the corresponding shifts in peak positions (left shift for 6.5% and right shift for −3.9%), as shown in Fig. 11 ▸(*e*). Therefore, image distortion such as ellipticity must be corrected before applying the PDF conversion to avoid any misleading PDF results due the acquisition and image quality.

Hart (2017 ▸) made a detailed study of the image distortion problem in his PhD thesis, although no algorithm was proposed for distortion correction. He stated that Abbé’s equation (Köhler, 1981 ▸) describes the diffraction-limited resolution of an optical microscope in the absence of distortions and aberrations, and consequently the practical extent of resolution will be limited if any distortions or aberrations are present.

The software *CRISP* (Hovmöller, 1992 ▸) addressed the distortion issue which may arise from electron lens defects, but the function of elliptical correction was not implemented in its early version. However, a scheme was suggested for correcting for the distortions by applying a mathematical function which would be the inverse of the distortions.

The observed circular distortion in an EDP could be suppressed before recording the pattern by tuning the imaging and diffraction astigmatism values, as suggested by Mitchell (2022 ▸), or by correcting the lens distortion in the transmission electron microscope by following the method proposed by Capitani *et al.* (2006 ▸). Post-correction of the distortion can be done by image analysis using curve fitting with an ellipse function in *r*–θ space (Hou & Li, 2008 ▸), or with the sine-wave function (Capitani *et al.*, 2006 ▸), on the condition that the diffraction pattern has been spread linearly in azimuth. The current work makes use of sine-wave function fitting on azimuthally spread electron diffraction. The sine-wave function is defined as

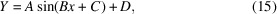

where the four parameters *A*, *B*, *C* and *D* define the sine-wave curve. *B* is equal to 2 as the period is 2π. *D* is the non-distorted circle radius, which is known if the crystal structure of the tested sample is known. Only two parameters, *A* and *C*, need least-squares fitting to extract the distortion axis *C* in azimuthal angle by finding the minimal sum of the square of deviations,

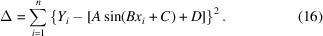

Accordingly, the distortion ratio along the long axis ɛ_*a*_ = (*D* + *A*)/*D* and that along the short axis ɛ_*b*_ = (*D* − *A*)/*D*. With the extracted elliptical axis direction (*C*) and deformation ratio (ɛ_*a*_ and ɛ_*b*_), a distortion-free image **I**′(*x*, *y*) can be created from the raw image **I**(*x*, *y*) by following the conversion of the matrices of rotation **R** and shearing **S**,

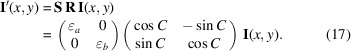




**Figure 12:**
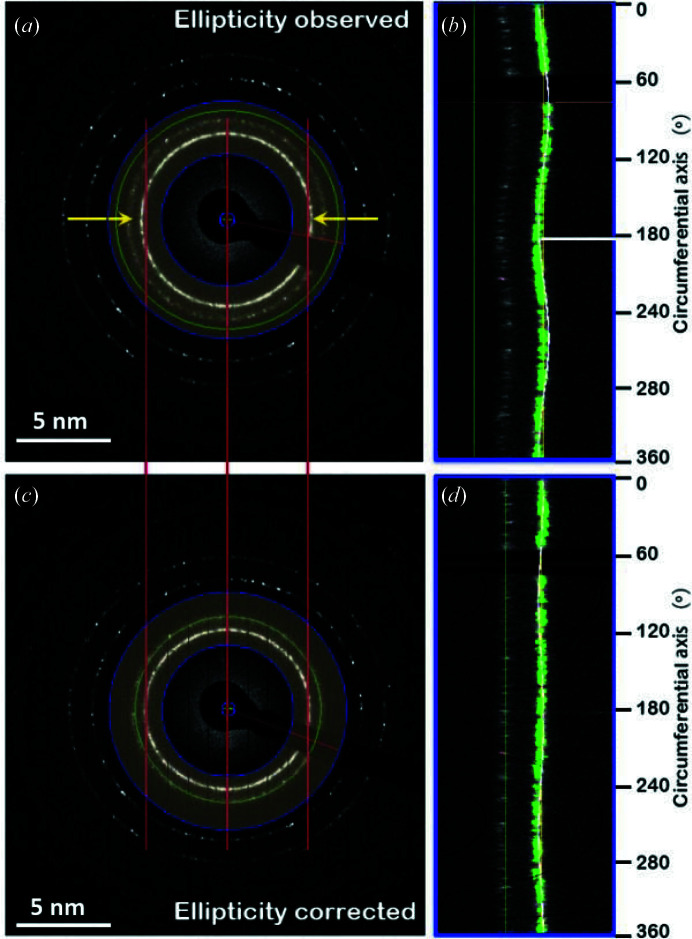
An investigation of the ellipticity of an EDP using a least-squares fitting algorithm. (*a*) The experimental EDP of an Au sample showing horizontal elongation. (*b*) Azimuthal spreading of the region of interest, highlighted in the circular band in panel (*a*). The least-squares fit gives a distortion direction close to 180°, which is identical to the observed horizontal direction. (*c*) The recreated EDP pattern after correction of the detected distortion. (*d*) Azimuthal spreading of the region of interest highlighted in the circular band in panel (*c*), showing that the ellipticity is nearly cancelled.

To demonstrate the efficiency of the above method for ellipticity correction on an EDP, Fig. 12 ▸ shows a comparison of an EDP before and after correction of ellipticity using a least-squares fitting algorithm. Fig. 12 ▸(*a*) is an experimental EDP obtained on the nanoparticle Au sample, where elongation is observed along the horizontal direction marked with two arrows and two vertical lines shared by the two EDPs. The light-blue semi-transparent circular band of the elliptical EDP in Fig. 12 ▸(*a*) is azimuthally spread along the circumferential direction in an angular range of (0°, 360°). An apparent sine-wave profile of the diffraction ring as shown in Fig. 12 ▸(*b*) indicates that the expansion direction points to an angular position close to 180°. Combined with the amplitude of the sine wave, we can make a correction of the observed elliptical pattern and recreate a distortion-corrected EDP which is shown in Fig. 12 ▸(*c*). The azimuthal spread of the same circular band of the distortion-corrected EDP successfully gives a straight line corresponding to the diffraction ring [Fig. 12 ▸(*d*)].

## 8. Conclusions

The *EDP2PDF* software for extracting PDFs from EDPs has been developed for automated structure analysis for materials science. This program provides robust background subtraction from the extracted diffraction intensity profile and fully automatic conversion of the EDP into a PDF without requiring external software. The input can come from either a diffraction intensity profile or an EDP.

## Supplementary Material

Zipped file containing manual. DOI: 10.1107/S1600576723004053/te5109sup1.zip

## Figures and Tables

**Table 1:** PDF peak positions and coordination number (CN) for crystalline Au

	Peak position						
	1	2	3	4	5	6	7
Distance (Å)	2.80	4.04	4.97	5.76	6.41	7.05	8.63
Direction	[110]/2	[100]	[112]/2	[110]	[310]/2	[111]	[411]/2, [330]/2
CN	12	6	24	12	24	8	36

## Appendix A Scattering vector

To convert to the structure function, the scattering vector magnitude *Q* must be derived from the semi-angle θ by the equation

Here,  is the characteristic angle for an energy loss Δ*E*. The inelastic error is more significant for electrons of high energy loss, although such electrons can be excluded using an energy filter (Cockayne & McKenzie, 1988 ▸). Since electron diffraction is assumed to be filtered as zero-loss peak diffraction, this component  can be regarded as zero. Consequently, the scattering vector magnitude has the form

## Appendix B Average atomic number density

To calculate the average atomic number density, we consider equation (8), the relationship between the scattering vector *Q* and *s*. The PDF can have another form when using *s* as the scattering vector magnitude instead of *Q*,

This is equivalent to equation (2) of Mitchell & Petersen (2012 ▸). However, equation (20) will induce a terminal ripple at *s* = *s*_min_ and when *s* extends into the negative regime. When *r* is below *r*_min_, where *r*_min_ is pre-known from the structure of the material (for instance, this value is 2.88 Å for gold with a face-centred cubic structure and lattice parameter 4.08 Å), the local atomic number density becomes zero. Consequently, the average atomic number density is obtained directly by linear fit of *G*(*r*) at its lowest part corresponding to *r* ≤ *r*_min_. This will give

where *G*(*r*)′ is the linearly fitted result of *G*(*r*) when *r* ≤ *r*_min_. This linear fitting method gives an accurate average atomic number density when the minimum interatomic distance *r*_min_ is known, although it is often unknown. The value of *r*_min_ can be approximated as 1 Å to maintain the physical meaning when the exact shortest interatomic distance of the investigated material is unknown. When it becomes difficult to estimate the distance *r*_min_, the following alternative method can be utilized.

An alternative to calculating the average atomic number density is through numerical estimation. In particular, we count the geometric average of the atomic number density for each element if the atomic percentage is known. For a given material with *N* elements, each of them has an atomic number *Z*(*r*) (*r* = 1 to *N*), molar mass *M*(*r*), density ρ(*r*) and atomic percentage *C*(*r*). The average atomic number density ρ is calculated by

where *N*_a_ is Avogadro’s number (6.022 × 10^23^).

However, there are two problems with this approach. Firstly, it is not always clear what percentage of each element is present in the investigated material, and secondly, the estimated result of the average atomic number density is not always accurate because of the chemical bonding status, the status of the elements, and the strain or thermal distribution.

## Appendix C Average coordination number

The average coordination number corresponding to an interatomic distance range is measured as the integration over the corresponding peak in the radial distribution function (Cockayne & McKenzie, 1988 ▸),

However, the integration result is not accurate unless the following considerations have been applied to the normalized PDF: background subtraction, energy filtering, average atomic number density ρ, minimum interatomic distance *r*_min_ and calibration.
