# Supplementary material for: EDP2PDF: a computer program for extracting a pair distribution function from an electron diffraction pattern for the structural analysis of materials
Source: J Appl Crystallogr. 2023 May 31;56(Pt 3):889–902. doi: 10.1107/S1600576723004053 (PMC10241047; doi:10.1107/S1600576723004053)
Supplement: Supplementary file 1 [file j-56-00889-sup1.zip › EDP2PDF-Ver 1.0 - Manual.pdf]

EDP2PDF

Version 1.0

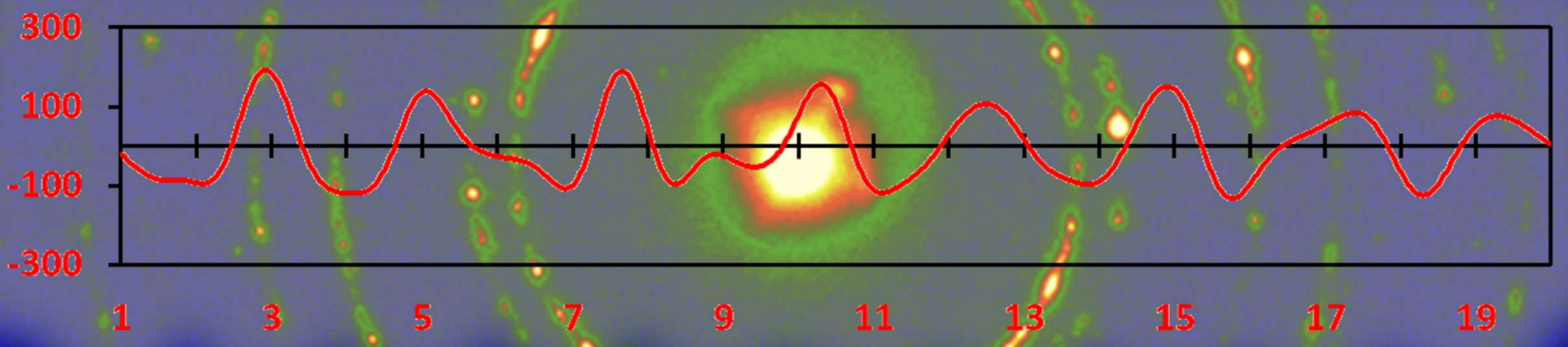

Dr Hongwei Liu

ACMM, The University of Sydney, The University of Sydney

2023.02.10

Instructions

- Specific description
- About the author
- Main functions
- Basic operation
- Acknowledgement

Specific description

Computer language:	Visual Basic 6.0
Core algorithm:	Machine vision
Knowledge background:	Crystal diffraction
Key technology:	Image and spectrum processing
Script Line number:	10, 000
Running environment：	OS: Windows 9X/2000/xp/7/10/11<br>Inner memory: 64 MB<br>Hard disk space: 50 MB

About the Author

- Name: Hongwei Liu
- Research field: Advanced materials preparing and characterization
- Affiliation: ACMM, The University of Sydney
- Email: hongwei.liu@Sydney.edu.au

Main functions

- Convert electron diffraction pattern (EDP) and X-ray diffraction pattern (XRD) into pair distribution function pattern (PDF) both in auto mode and manual mode
- Work for single or multiple crystalline crystal electron diffraction pattern images and XRD two column TXT format spectrum.
- Work for non-complete electron diffraction pattern

- Image processing for electron diffraction pattern
  - Histogram
  - Contrast inversion
  - Smoothing
  - Sharpening
  - Edge searching
  - Binarization
  - Scale bar rectangular shape searching
  - Circle centre searching
- Spectrum processing for X-ray diffraction pattern
  - Smoothing
  - Background subtraction

- Conversion parameters can be adjusted as per requirement
- Step-to-step conversion for non-complete election diffraction pattern
- One-key conversion is available if scale bar information is correct (F1 for EDP2XRD and F2 for EDP2PDF)
- Generated X-ray pattern and PDF pattern can be exported to TXT format
- Crystalline information file (CIF) can be imported followed by d-spacing and diffraction angle calculation which is useful for peak searching and indexing
- Chemical information can be input manually followed by saving as TXT format or import from a saved TXT file.

Operation

1. Set conversion parameters：

—Input reciprocal scale bar (defaulted as 5)，unit is 1/nm;

—Input X-ray wavelength (defaulted as 0.154)， unit is nm;

—Input normal resolution (defaulted as 0.1)， unit is deg;

—Input radius resolution (defaulted as 0.1)， unit is deg;

—Input start angle (defaulted as 15)， unit is deg.

Remark: one-key conversion is available if all the above parameter have been set correctly. F1 converts into XRD and F2 into PDF.

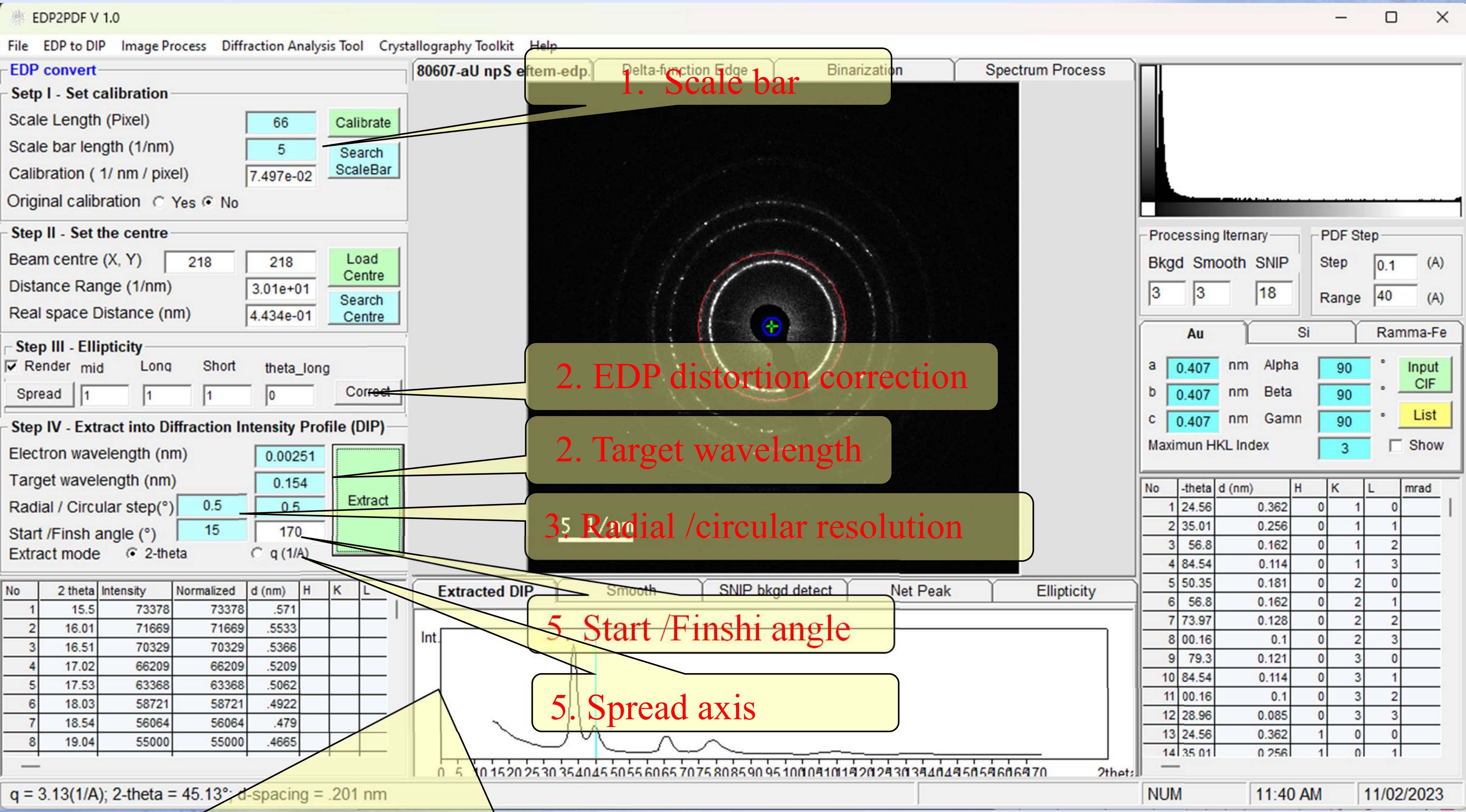

Mouse right click to a peak to automatic index the reflection hkl plane when using extract mode 2-theta

2. Open an image of electron diffraction pattern

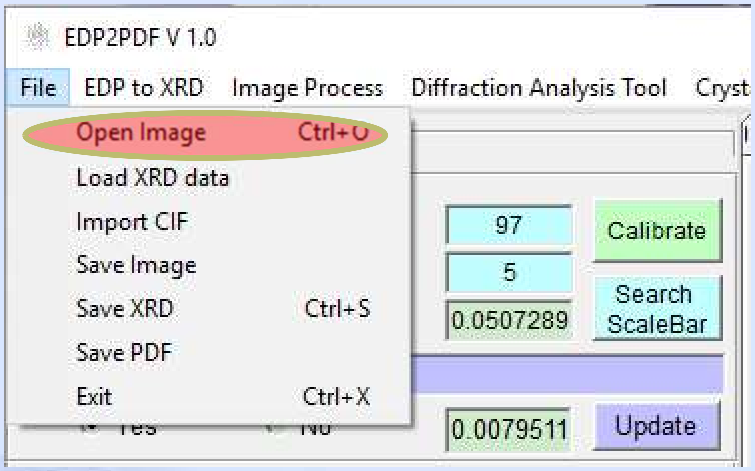

3. Input calibration data

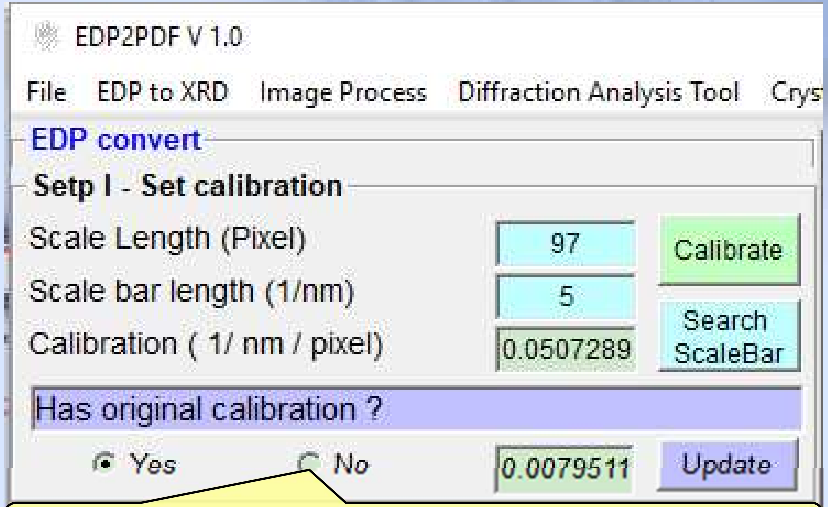

Click "No" is no calibration data is available.

4. Input chemical composition in atomic percentage

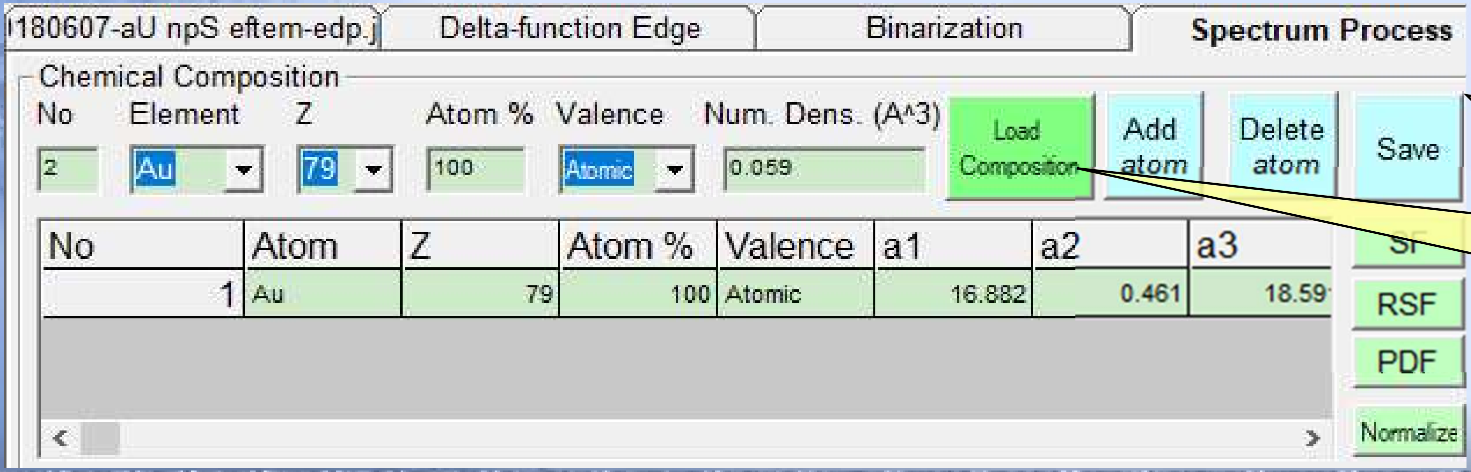

Chemical composition can be saved as TXT format for recall.

You can load a saved TXT format composition.

5. Input lattice parameters,

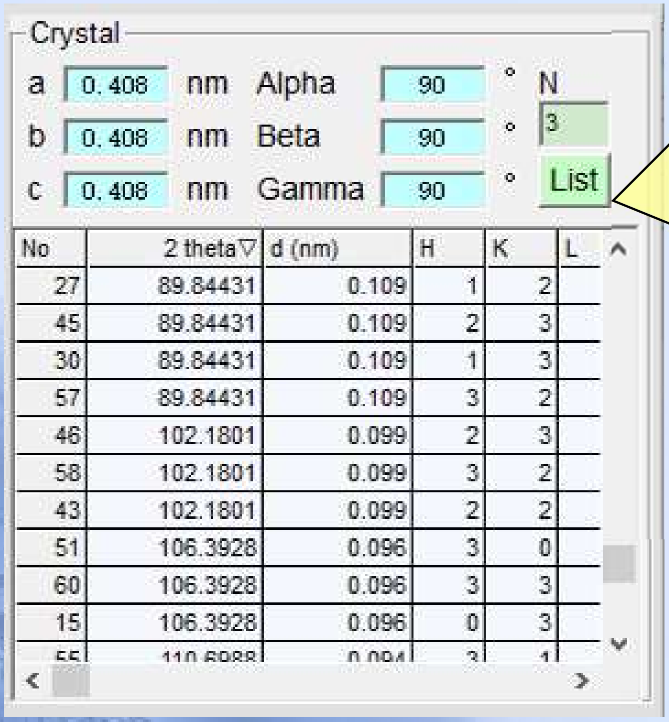

It can be any Bravais lattice. Skip if unknown

6. Input Conversion parameters

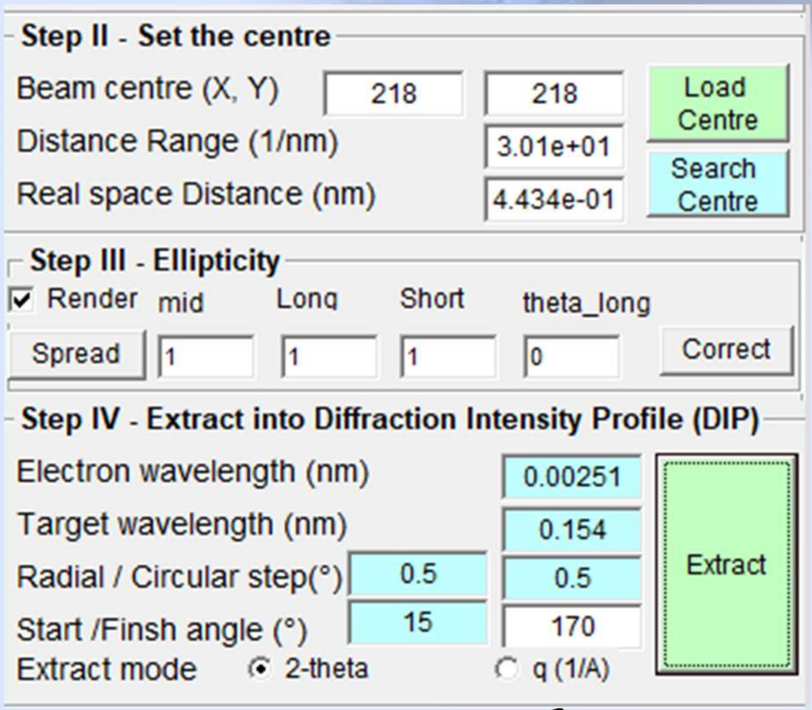

Smaller radius resolution leads to a lower deviation of the extracted XRD and PDF profiles.

7. Auto Mode：One-key conversion

- From Main menu → EDP to XRD → Simple XRD or Press F1
- Wait until conversion finished.

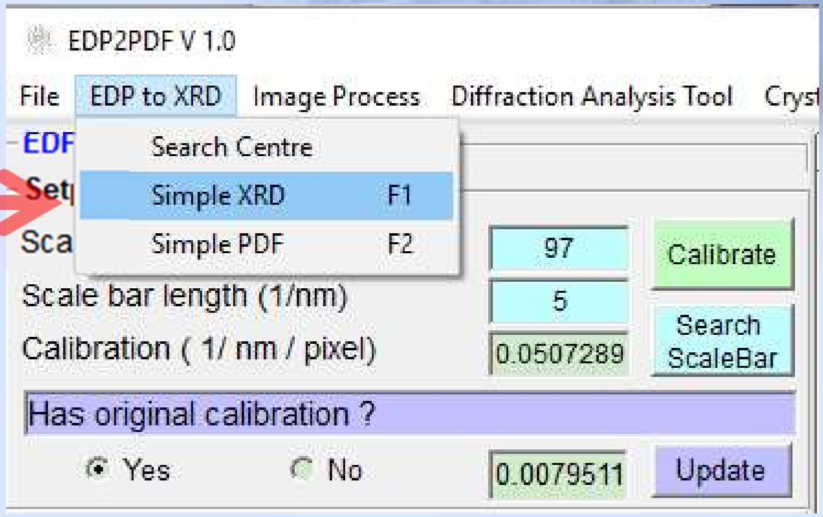

- From Main menu → EDP to PDF → Simle PDF or Press F2
- Wait until conversion finished.

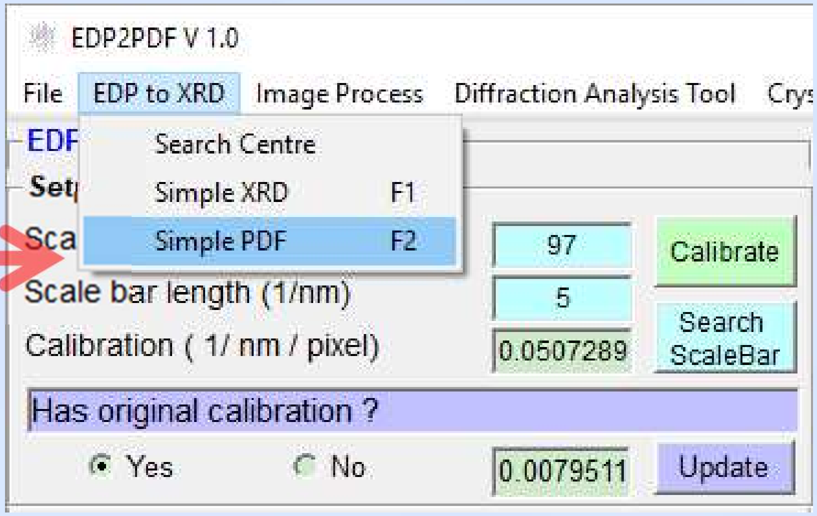

8. Conversion results

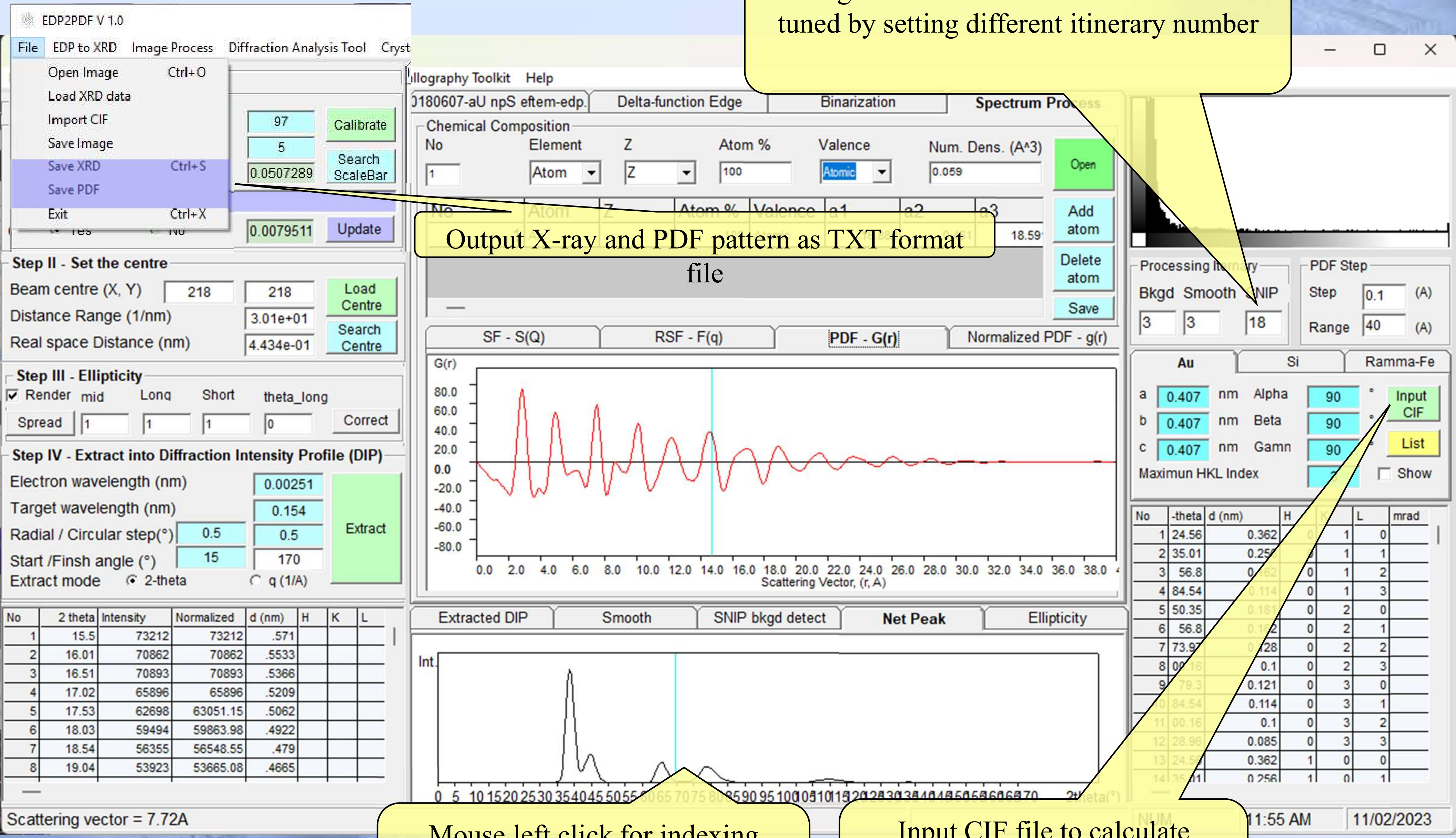

Background detection and removal can be tuned by setting different itinerary number

Output X-ray and PDF pattern as TXT format file

Mouse left click for indexing diffracted peaks according to CIF file if structural factor is known

Input CIF file to calculate planar d-spacing and diffraction angle 2-theta。

9. Manual Mode1: Simple conversion

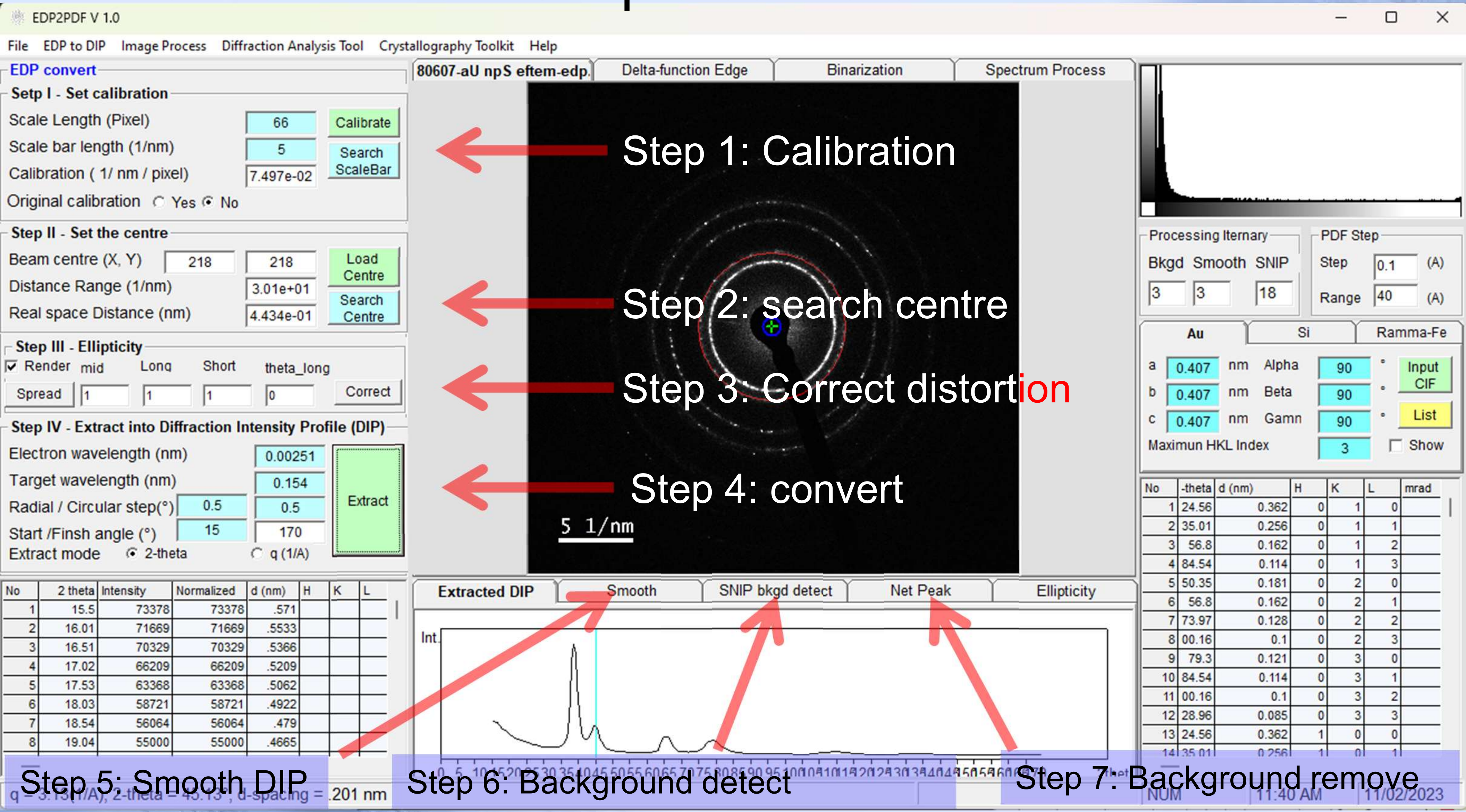

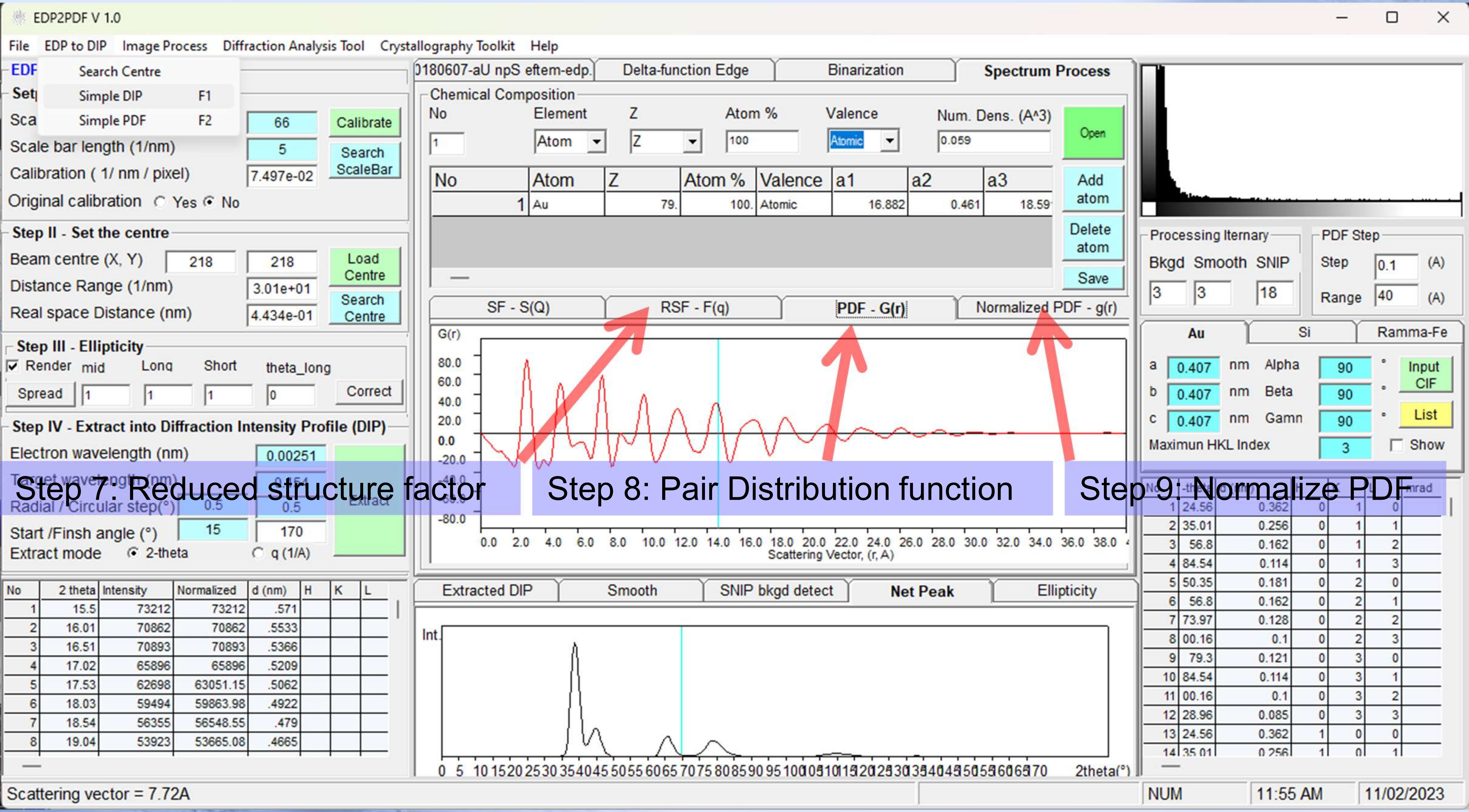

Manual mode simple conversion is similar as auto mode simple conversion but with the freedom of controlling each step of conversion.

10 Manual Mode 2: Manual conversion

Step 1: Click “Load centre”, enter scale bar definition mode.

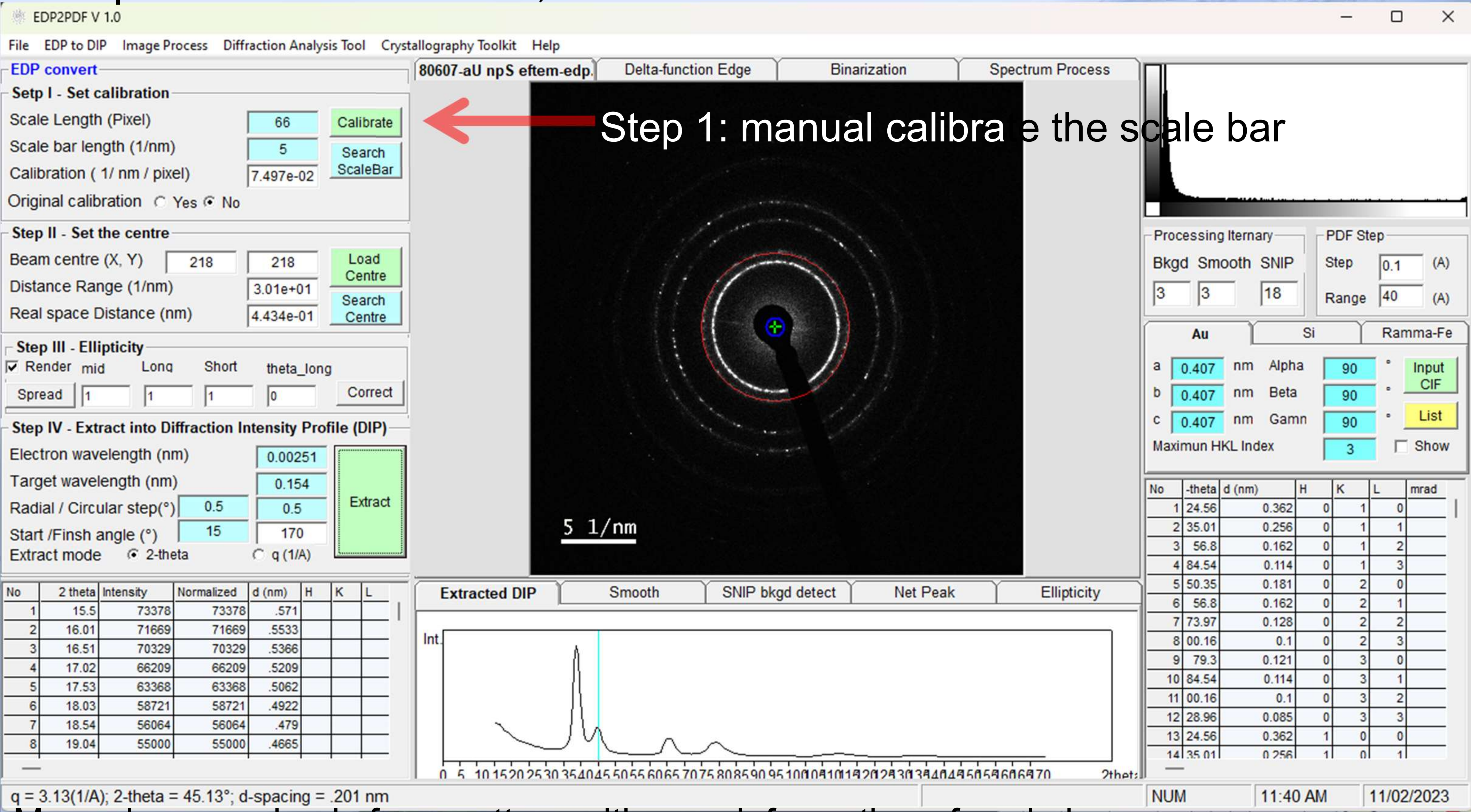

Manual conversion is for a pattern with poor information of scale bar.

Step 2: Define scale bar length

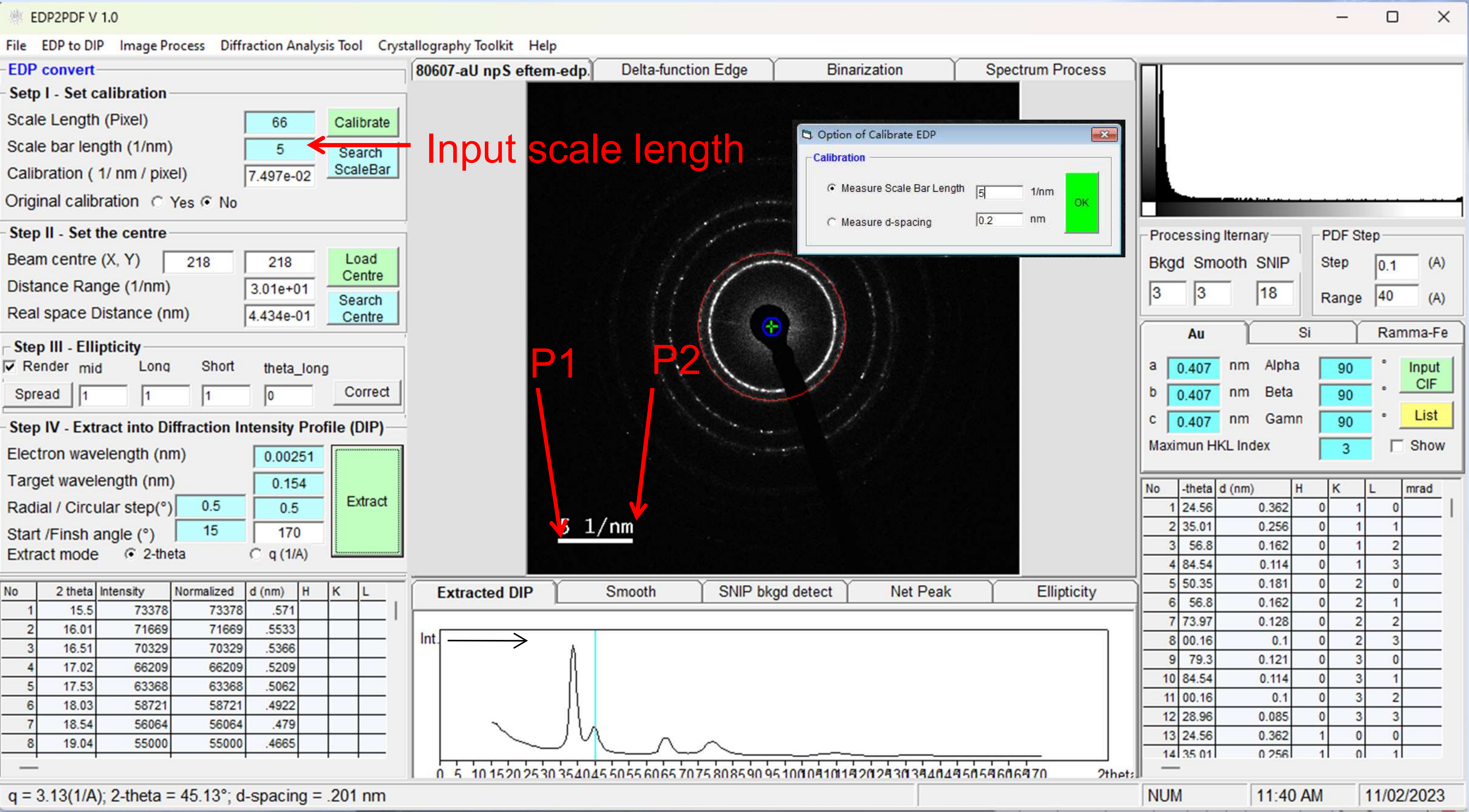

Mouse left key click P1 the start point of scale bar, Keep left key down and move to P2 the end point of scale bar, the release left key. Input the calibration value in the Dialogue box. The length of scale bar will be input into Scale length (Pixel) .

Step 3: Set diffraction ring centre

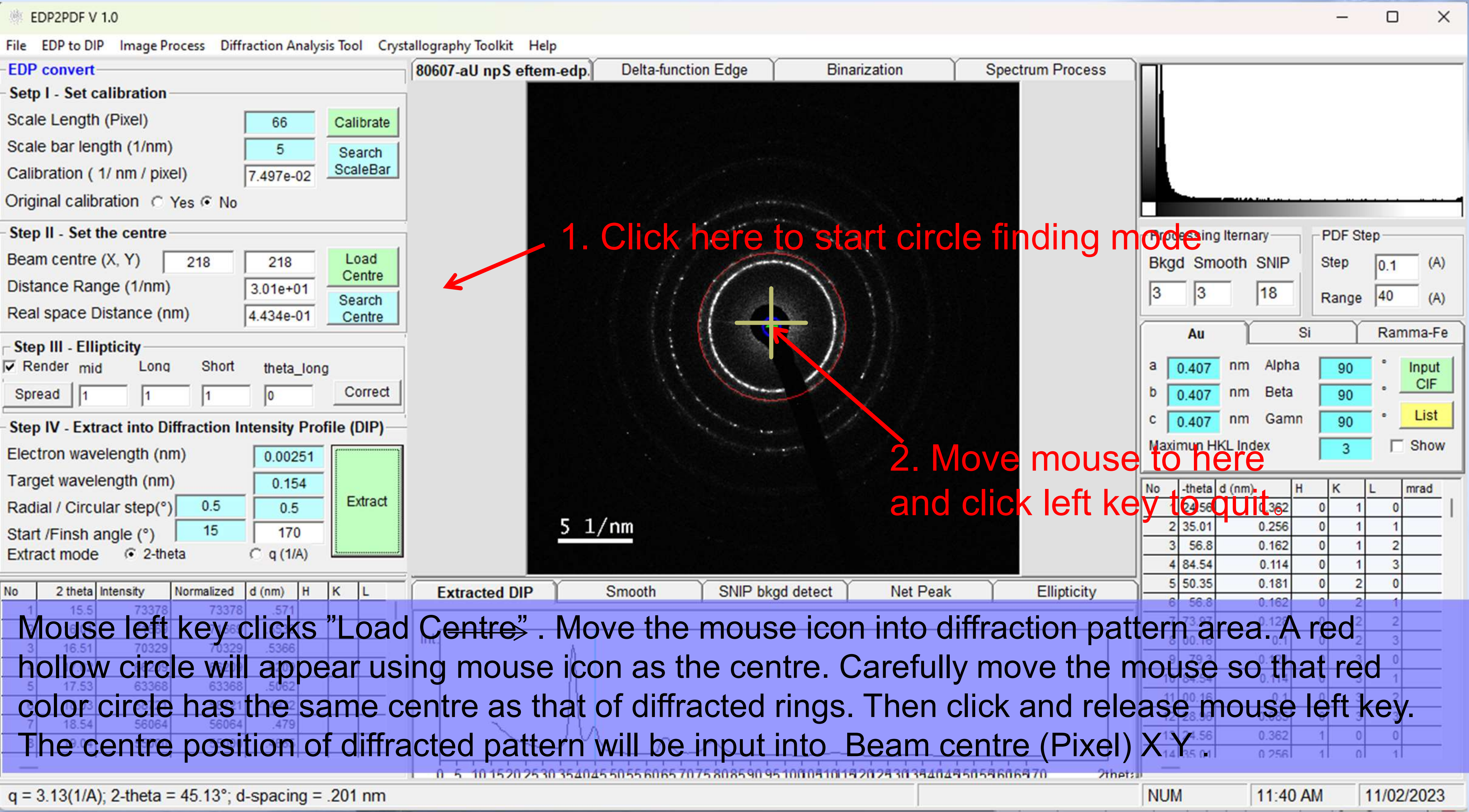

Mouse left key clicks "Load Centre" . Move the mouse icon into diffraction pattern area. A red hollow circle will appear using mouse icon as the centre. Carefully move the mouse so that red color circle has the same centre as that of diffracted rings. Then click and release mouse left key. The centre position of diffracted pattern will be input into Beam centre (Pixel) X Y .

Step 4: Click "Extract" and conversion starts.

Acknowledgements

Enlightening discussion with Dr Matthew Foley, Image processing and analysis specialist at The University of Sydney is greatly appreciated.

Dr Hongwei Liu
Sydney
2023.02.10

Tank you for choosing EDX2PDF!

Any comment is welcome.
